# Systems biology approaches to identify potential targets and inhibitors of the intestinal microbiota to treat depression

**DOI:** 10.1038/s41598-023-38444-8

**Published:** 2023-07-11

**Authors:** Fei Teng, Zhongwen Lu, Fei Gao, Jing Liang, Jiawen Li, Xuanhe Tian, Xianshuai Wang, Haowei Guan, Jin Wang

**Affiliations:** College of Traditional Chinese Medicine, Shandong University of Traditional Chinese Medicine, Jinan, 250014 China

**Keywords:** Databases, Gene regulatory networks, High-throughput screening, Statistical methods, Virtual drug screening, Drug discovery, Microbiology, Biomarkers

## Abstract

Depression is a common mental disease, with some patients exhibiting ideas and behaviors such as self-harm and suicide. The drugs currently used to treat depression have not achieved good results. It has been reported that metabolites produced by intestinal microbiota affect the development of depression. In this study, core targets and core compounds were screened by specific algorithms in the database, and three-dimensional structures of these compounds and proteins were simulated by molecular docking and molecular dynamics software to further study the influence of intestinal microbiota metabolites on the pathogenesis of depression. By analyzing the RMSD gyration radius and RMSF, it was finally determined that NR1H4 had the best binding effect with genistein. Finally, according to Lipinski's five rules, equol, genistein, quercetin and glycocholic acid were identified as effective drugs for the treatment of depression. In conclusion, the intestinal microbiota can affect the development of depression through the metabolites equol, genistein and quercetin, which act on the critical targets of DPP4, CYP3A4, EP300, MGAM and NR1H4.

## Introduction

Depression is a common neuropsychiatric disorder with a high recurrence rate. Different types of depression affect approximately 350 million people worldwide, and the prevalence of depression is increasing^1,2^. Currently, the incidence of depression is increasing yearly, causing considerable losses to families and society. According to the World Health Organization, the disease burden of depression will rank first in Western countries, and depression will become the fourth most significant disease in the world by 2030^3,4^. The etiology of depression is complex, the mechanism is still unclear, and the treatment response is insufficient, thereby reducing the efficiency of clinical diagnosis and treatment^5,6^. Therefore, it is critical to explore the etiologic mechanisms of depression. Selective serotonin reuptake inhibitors, serotonin, norepinephrine reuptake inhibitors, and other drugs are the primary treatment methods in modern medicine, but some patients do not achieve good efficacy and experience severe adverse reactions, such as cardiac toxicity^7^.

Ongoing research on this subject shows that the intestinal microbiota plays a crucial role in maintaining human health and preventing various diseases. The intestinal microbiota is also important for the normal development and maintenance of brain function and is related to various mental and neurological diseases^8,9^. The central nervous system can regulate the physiological activities of the intestine. In contrast, various physiological responses caused by changes in the composition of the intestinal microbiota can be transmitted to the brain and other central nerves to stimulate the intestinal microbiota, resulting in various physiological and pathological phenomena. Therefore, the “brain–gut” axis theory opens up a new avenue for treating depression^10,11^. Relevant studies have suggested that the intestinal microbiota of patients with depression differs significantly from that of people without depression and can affect brain function via the brain-gut axis, suggesting that the intestinal microbiota is closely related to the occurrence and progression of depression^12–14^. Many studies have shown that the microbiota–gut–brain (MGB) axis affects brain function and behavior; regulates stress, anxiety, and cognition; and causes depressive symptoms. Therefore, depression can be treated by regulating the intestinal microbiota^15–17^. Here, we explored the pathogenesis of depression based on the intestinal microbiota.

With the popularity of gene microarrays and RNA sequencing, bioinformatics has been widely used to analyze high-throughput sequencing data for various diseases. In this study, we used the Gene Expression Omnibus (GEO, https://www.ncbi.nlm.nih.gov/geo/) database and the GutMgene database (http://bio-annotation.cn/gutmgene/home.dhtml) to investigate the mechanism of depression and predict a promising drug for its treatment.

**Figure 1:**
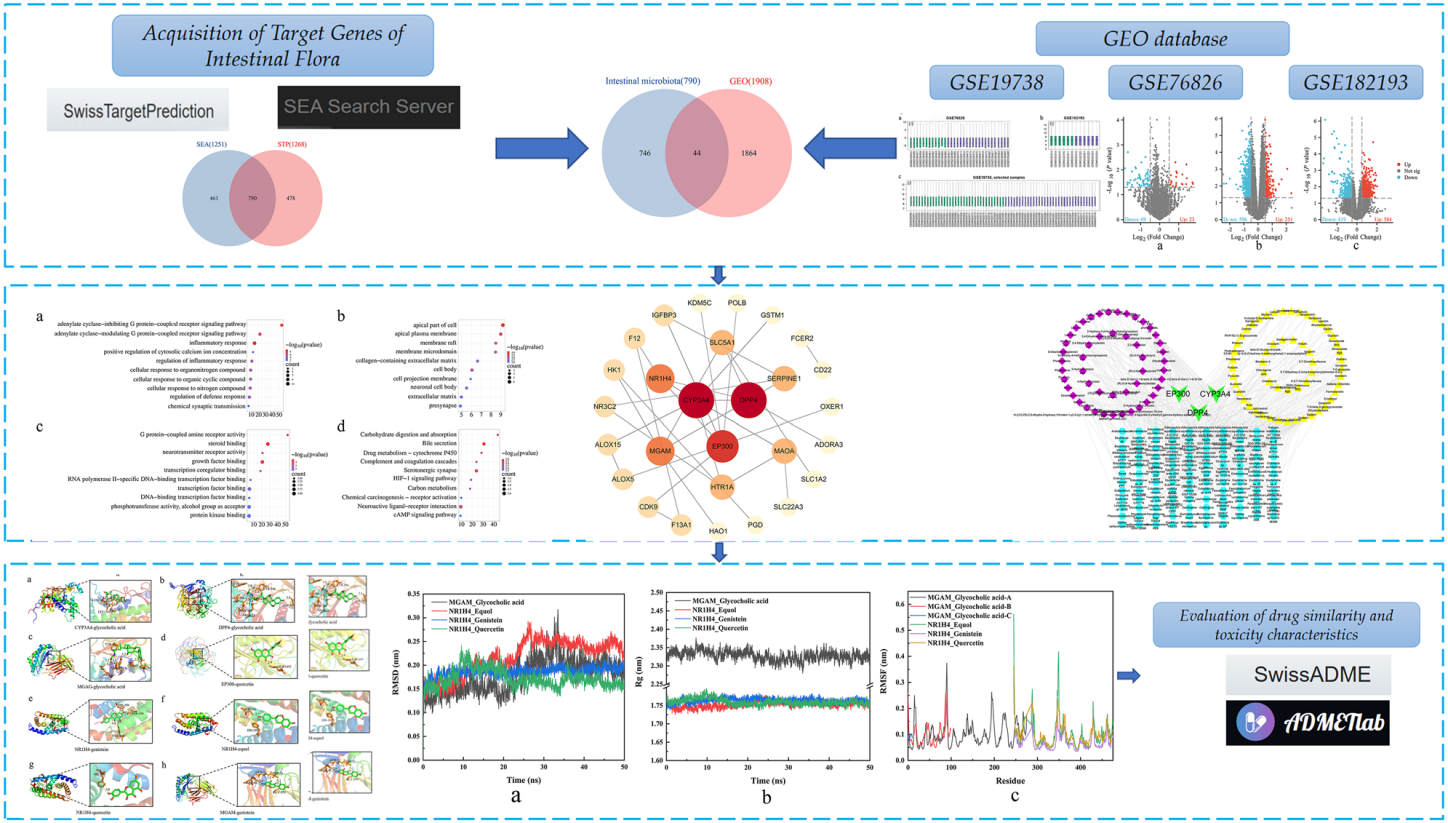
Technology roadmap for this study.

We obtained the intestinal microbiota and metabolite information from the human intestinal microbiota database. Then, we predicted the metabolite targets through two online target prediction websites, SEA and STP, and intersected the prediction results of the two websites to obtain the target genes of metabolites of intestinal microbiota and the potential effects on human diseases. Then, we selected three depression-related datasets from the GEO database, normalized them and used a volcano plot to show the differentially expressed genes in depression. The differentially expressed genes were intersected with the potential target genes of intestinal microbiota metabolites affecting human diseases, and the potential target genes of intestinal microbiota metabolites affecting depression were obtained. Subsequently, a protein‒protein interaction network and GO and KEGG enrichment analyses of these key differentially expressed genes were constructed, and the “intestinal microbiota–metabolites–target gene” network relationship was constructed. According to the network topology analysis by Cytoscape, we screened the key small-molecule compounds and proteins, carried out molecular docking and molecular dynamics simulation verification, and finally evaluated the potential small-molecule compounds for drug toxicity. The technology roadmap for this study is presented in Fig. 1.

## Materials and methods

### Acquisition of target genes of intestinal microbiota

Metabolite and target gene information for the intestinal microbiota and microflora were extracted from GutMgene, a database of intestinal microbiota. Using the similarity ensemble approach (SEA) (https://sea.bkslab.org/) and the SwissTargetPrediction (STP) (http://www.swisstargetprediction.ch/) database, we mined metabolites that may influence target genes and took the genes at the intersection of the two methods as intestinal microbiota target genes^18–20^.

### Download and standardization of microarray data and identification of differentially expressed genes (DEGs)

Search strategies included “depression” [MeSH term] or “depression” [all fields]; “Homo sapiens” [porgn]; and “expression analysis by array” [filter].

According to the above retrieval strategies, the expression profiles of three gene sets, GSE19738, GSE76826, and GSE182193, were selected from the GEO database for analysis. After retrieving and standardizing the three datasets using the GEOquery package in the R language (version 4.1.3), boxplots of the gene expression data were designed using the ggplot2 package^21,22^. Genes with a threshold standard of |log FC > 0.5| and *p* value < 0.05 were selected as DEGs, and SAT samples were screened using a linear model of microarray data packets. The Pheat map, ggplot2, and RColorBrewer packages were used to create DEG volcano maps.

### Identification of DEGs

The gene expression profiles of GSE19738, GSE76826, and GSE182193 were selected from the GEO database for analysis. DEGs were selected from the above three gene expression spectra, with threshold criteria of |logFC|> 0.5 and *p* < 0.05. After the duplicated DEGs were summarized and deleted, 44 differential genes were obtained from the intersection of DEGs and the target genes of intestinal microbiota. The 44 differentially expressed genes obtained through the intersection can be considered key genes that cause depression through the action of intestinal microbiota metabolites on target genes.

### Enrichment analysis of gene ontology (GO) and Kyoto encyclopedia of genes and genomes (KEGG) pathways

GO and KEGG enrichment analyses were performed on the selected 44 differentially expressed genes^23^. GO analysis annotates genetic information based on three aspects: molecular function, biological process, and cellular composition.

### Establishment of protein‒protein interaction (PPI) networks and identification of hub genes

The 44 differentially expressed genes were imported into the STRING database (https://string-db.org/), and the protein interaction relationship was obtained after hiding the free nodes. The results were imported into Cytoscape 3.7.2 for visualization, and network topology analysis was performed using the CytoNCA function to identify the key target genes^24,25^.

### Construction of the “intestinal microbiota–metabolites–target genes” network relationship

We used Cytoscape 3.7.2 to construct a "microbiota–metabolites–target genes" network to help determine the pharmacological mechanism. The degree value reflects the importance of nodes in the network. The higher the value, the more influential the node is. The core metabolites were identify by degree.

### Molecular docking

The core metabolites were downloaded from the PubChem database. The small molecules were hydrogenated, and the charge was calculated using Autodock 4.2.6 software^26^. The core target structures were obtained using the AlphaFold Protein Structure Database^27,28^; they were then imported into Autodock, and an appropriate grid box was set for molecular docking. Finally, the conformation with the lowest docking binding energy was chosen as the final docking result, which was visualized using PyMOL. If the docking binding energy is less than − 5 kcal mol^−1^, the receptor and ligand can bind spontaneously. The lower the binding energy is, the greater the possibility of binding, the more stable the binding conformation, and the greater the possibility of reaction. We set the appropriate grid box and we presented the the values as follows: DPP4 (x = 40, y = 45, z = 40), CYP3A4 (x = 56, y = 43, z = 56), EP300 (x = 41, y = 45, z = 43), MGAM (x = 37, y = 37, z = 36), NR1H4 (x = 30, y = 40, z = 43).

### Molecular dynamics simulation

Molecular dynamics (MD) simulation of the ligand‒receptor docked complex was carried out using GROMACS (version 2021.2)^29^. The protein topology file was generated using the AMBER99SB-ILDN force field^30^, whereas the ligand topology file was generated by the AnteChamber PYthon Parser interfacE (ACPYPE) script using the AMBER force field. MD simulations were carried out in a triclinic box filled with TIP3 water molecules and periodic bounding conditions. The system was neutralized with NaCl counter ions. Before MD simulation, the complex was minimized for 1000 steps and equilibrated by running canonical ensemble, constant-pressure (NVT) and constant-temperature (NPT) for 100 ps. Then, MD simulation was performed for 100 ns for each system under periodic boundary conditions at 310 K temperature and 1.0 bar pressure.

### Drug similarity and toxicity profile assessment

Similarities and toxicity characteristics were determined using SwissADME^31^ (http://www.swissadme.ch/) and validation ADMETlab^32^ network tools (https://admetmesh.scbdd.com/). Because these two factors are critical in the promotion of new agents, we evaluated their physicochemical properties and side effects.

## Results

### Intersection of the Venn diagram

**Figure 2:**
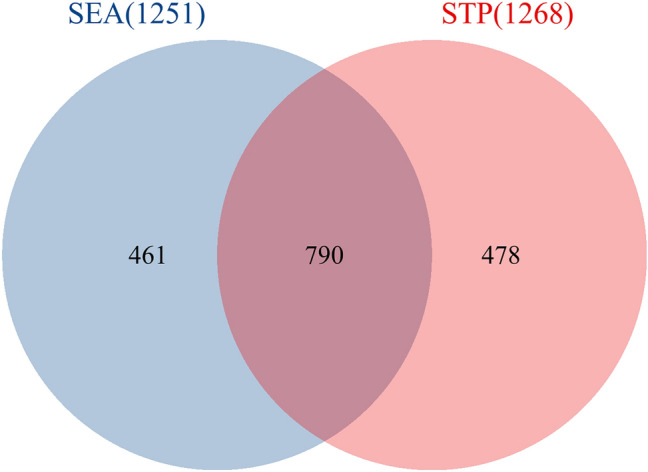
Acquisition of intestinal microbiota genes. There were 790 common targets between SEA (1251) and STP (1268).

After obtaining human intestinal microbiota data from the GutMgene database, the target genes that metabolites could act on were predicted via the SEA and STP databases (Tables S1 and S2, Supplemental Digital Contents, which demonstrate the prediction results of SEA and STP). A total of 790 targets were obtained by the intersection of the genes obtained from the two databases (Fig. 2) (Table S3, Supplemental Digital Content, which demonstrates the result of the intersection of the STP and SEA results).

### Identification of DEGs in depression

**Figure 3:**
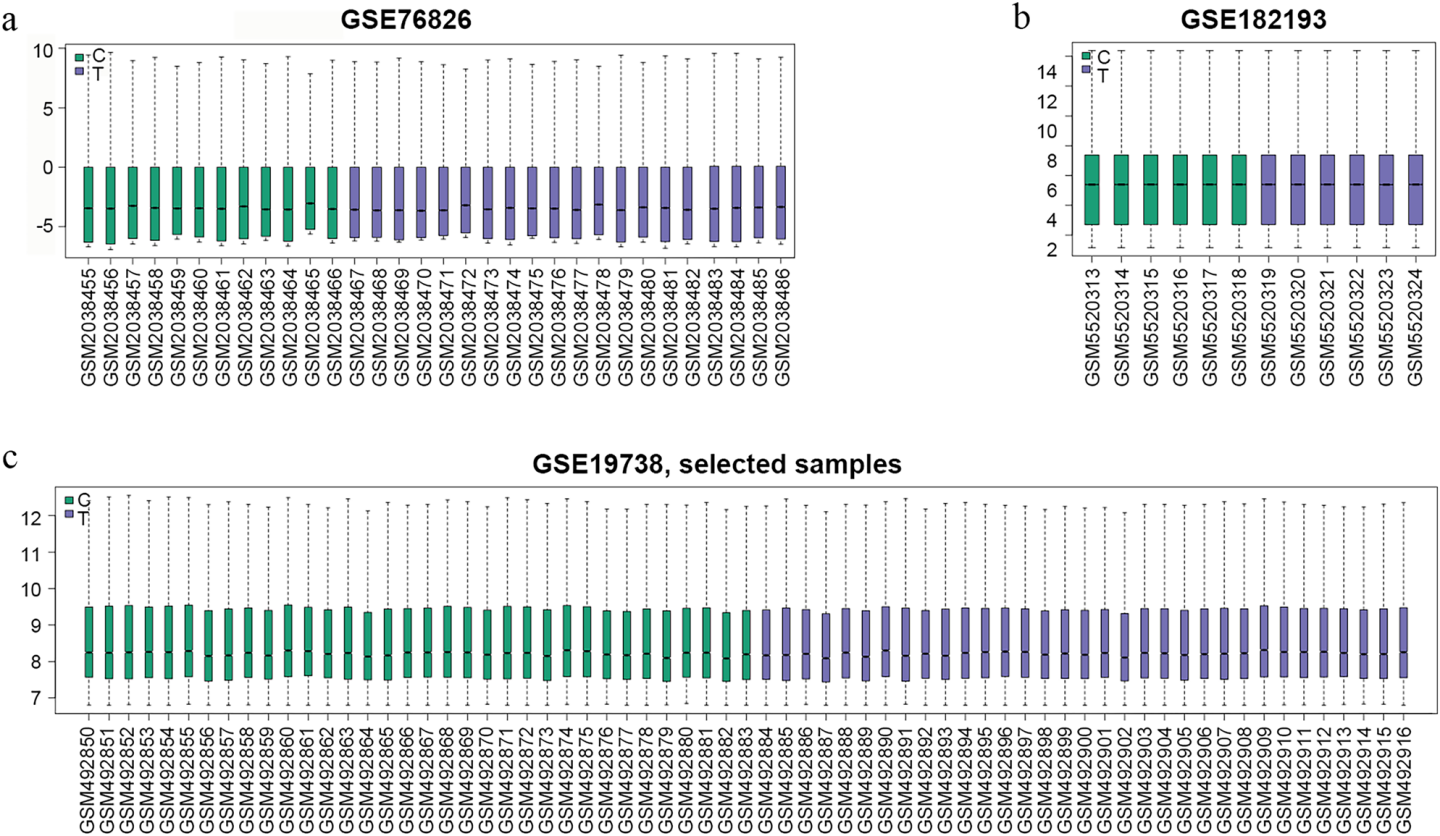
Boxplot of expression profiles after merging and standardization. The x-axis represents the sample symbol, and the y-axis represents gene expression values. The black line in the boxplot shows the median gene expression. (**a**) Standardization of GSE19738. (**b**) Standardization of GSE76826. (**c**) Standardization of GSE182193.

**Figure 4:**
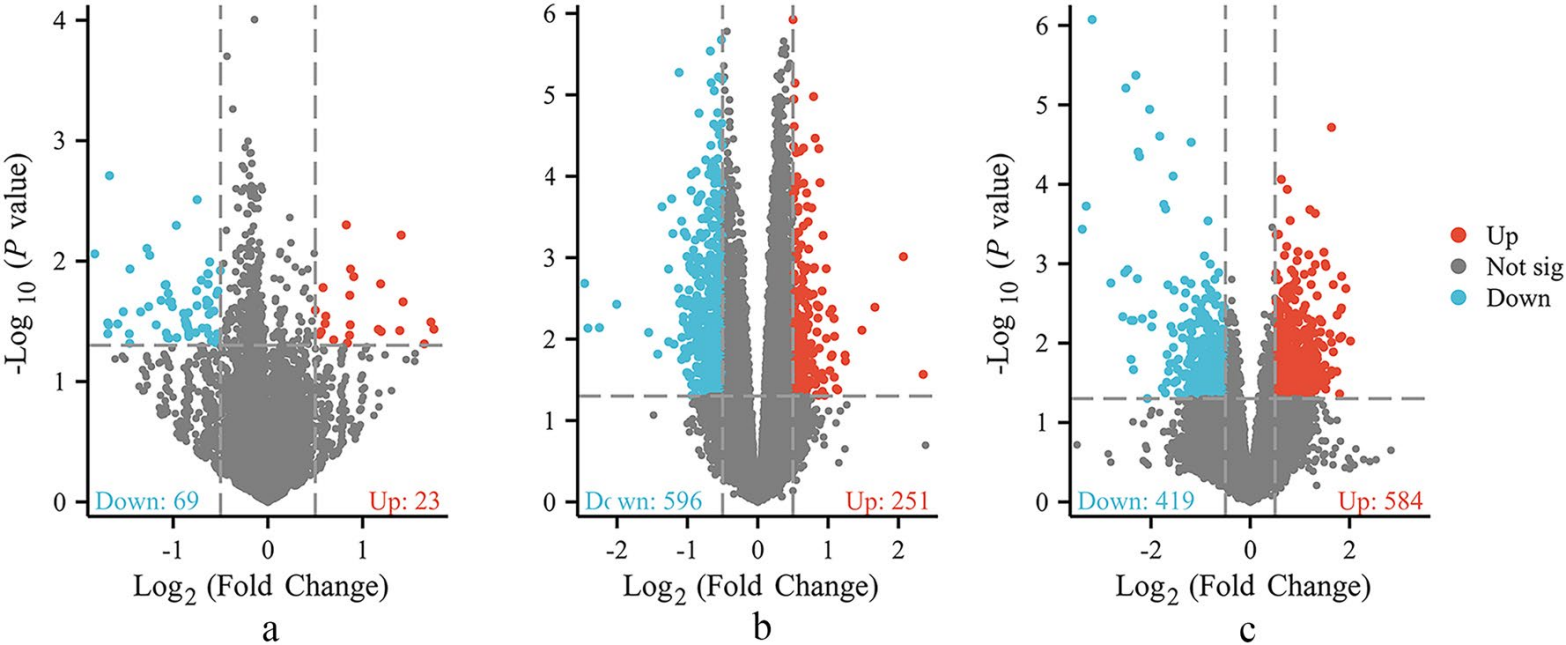
Volcano map used to identify DEGs. (**a**) GSE19738, (**b**) GSE76826, and (**c**) GSE182193. The abscissa in the volcano plot is the log2 (fold change) value, and the ordinate is the log10 (*p* value). The red dots represent upregulated genes, and the blue dots represent downregulated genes.

After dataset standardization **(**Fig. 3), 1942 DEGs were selected (|logFC|> 0.5, *p* < 0.05) based on the methods described above. The results included 858 upregulated genes and 1084 downregulated genes. Next, 1908 DEGs were reconstructed by merging and removing duplicates (Fig. 4) (Table S4, Supplemental Digital Content, which demonstrates differential genes after GEO dataset processing**)**.

### Intersection between the target genes of the intestinal microbiota and depression in the GEO database

**Figure 5:**
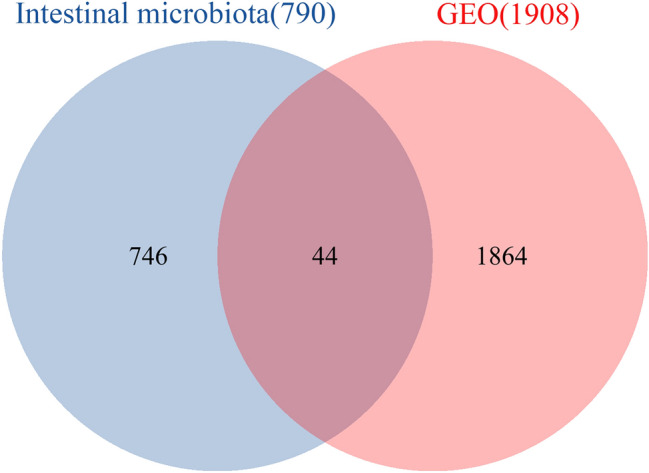
Intersection between target genes of the intestinal microbiota and depression in the GEO database.

Seven hundred ninety metabolite target genes were intersected with 1908 depression-related differentially expressed genes screened in the GEO database. Accordingly, 44 differentially expressed genes were obtained, which can be considered the crucial genes of intestinal microbiota metabolites that affect depression (Fig. 5) (Table S5, Supplemental Digital Content, which demonstrates the intersection of metabolite targets and GEO data).

### GO enrichment analysis and KEGG analysis of core genes

**Figure 6:**
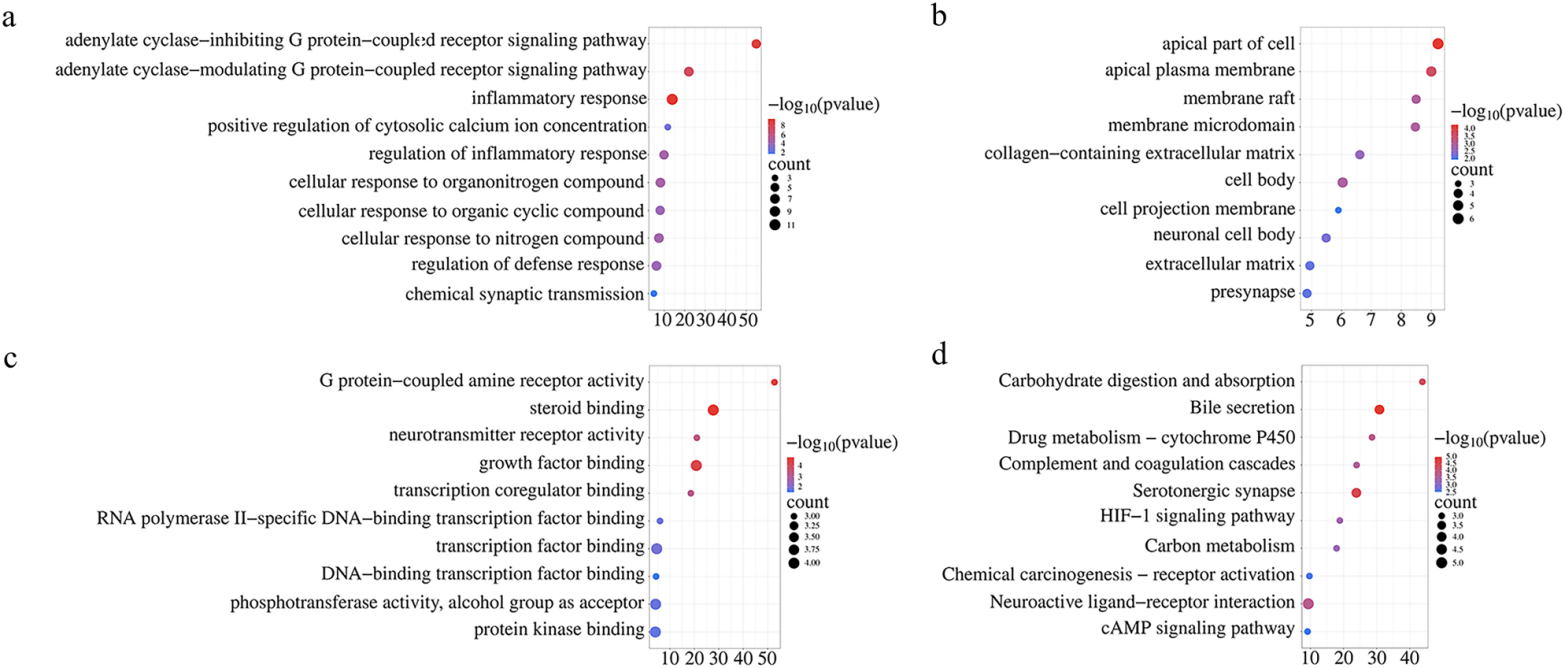
Enrichment analysis of GO and KEGG biological functions based on depression-related hub genes. (**a**) Molecular functions (MF); (**b**) biological processes (BP); (**c**) cellular components (CC); (**d**) KEGG.

The 44 differentially expressed genes were analyzed and visualized by GO and KEGG analyses based on the Metascape database (Fig. 6).

### Construction and analysis of the PPI network

**Figure 7:**
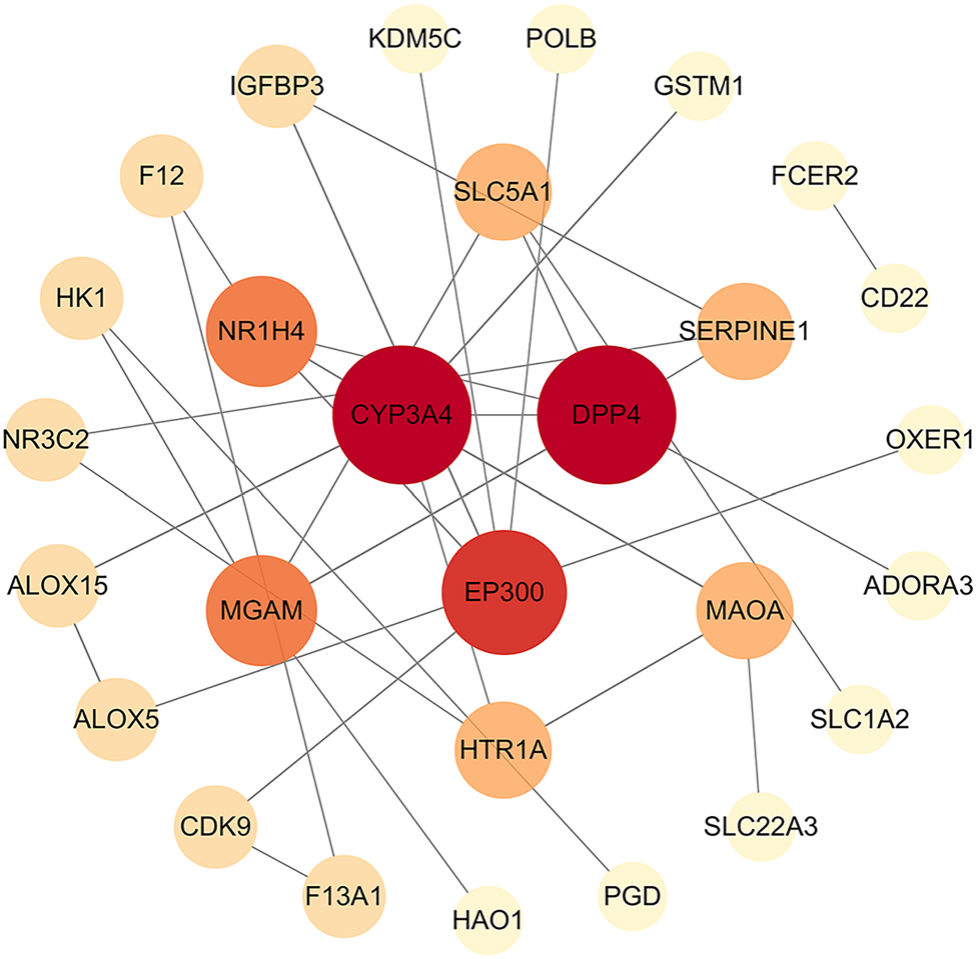
PPI network. Node size and color shade reflect the importance of the node.

The PPI network consisted of 28 nodes and 32 edges. Dipeptidyl peptidase 4 (DPP4), cytochrome P450 3A4 (CYP3A4), histone acetyltransferase p300 (EP300), maltase-glucoamylase (MGAM) and bile acid receptor (NR1H4) were identified as core targets according to degree values (Fig. 7) (Table S6, Supplemental Digital Content, which demonstrates the PPI network topology analysis results).

### Construction of the “intestinal microbiota–metabolites–target genes” network relationship

**Figure 8:**
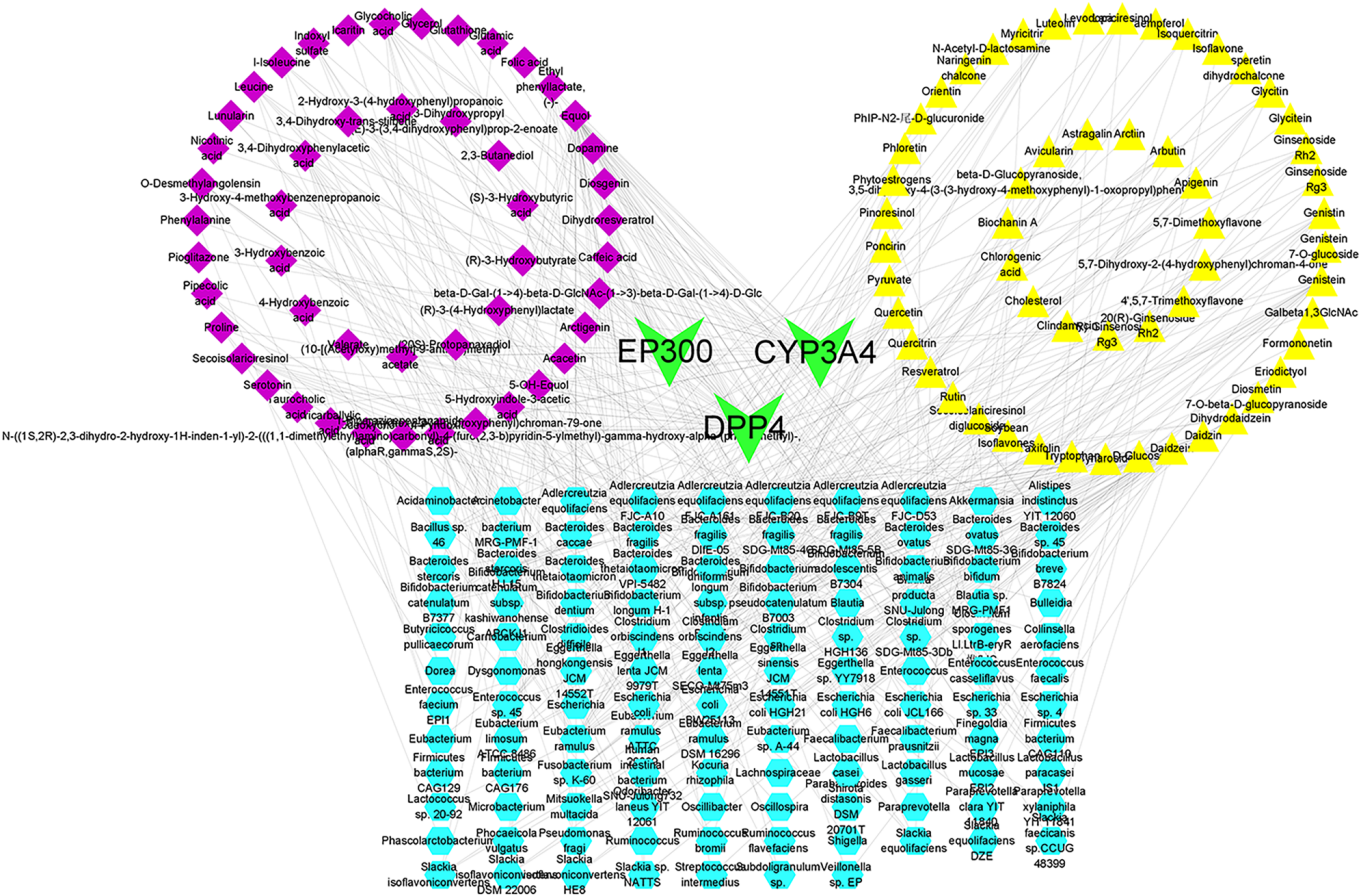
“Intestinal microbiota–metabolites–substrate–genes” network. Green represents target genes; blue represents the intestinal microbiota; purple represents metabolites; and yellow represents the substrate.

The network of "intestinal microbiota–metabolites–target genes" was constructed (Table S7, Supplemental Digital Content, which illustrates the intestinal microbiome–metabolite–substrate–target gene network). According to the degree values, equol, genistein, quercetin and glycocholic acid were considered the key metabolites affecting depression (Fig. 8).

### Molecular docking results

**Figure 9:**
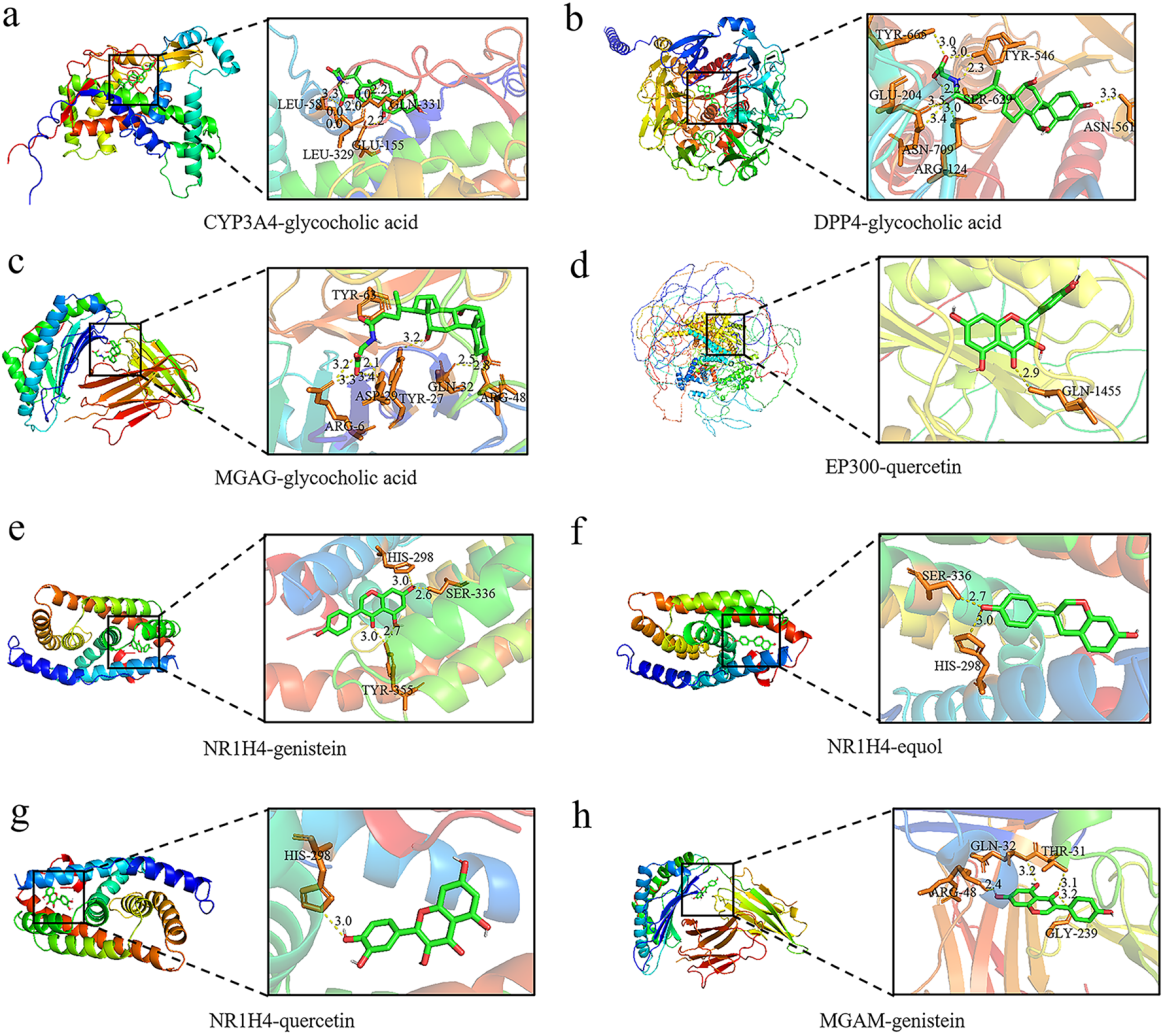
Molecular docking demonstration of core metabolites and core targets.

The key metabolites screened were molecularly docked with depression-related targets. The binding energies of the main active constituents and main targets were all < − 7.0 kcal/mol (Table S8, Supplemental Digital Content, which demonstrates the molecular docking results). The smaller the binding energy is, the higher the binding activity is, and the easier the compound is to bind to the target. Our molecular docking results show that key genes and metabolites can form stable conformations. Among them, the key gene MGAM had the best molecular docking results with the metabolite glycocholic acid, and its value was − 9.7 kcal/mol (Table 1). All molecular docking results were visualized (Fig. 9).

**Table 1:** Molecular docking results of core metabolites and core targets.

Protein	UniProt ID	Ligand	PubChem ID	Binding energy (kcal/mol)	Hydrogen bond interactions amino acid residue
DPP4	A0A7I2V2X8	Equol	91,469	− 7.4	TYR-546,ARG-357
DPP4	A0A7I2V2X8	Genistein	5,280,961	− 7.3	GLU-204,ARG-357,PHE-356,ARG-355
DPP4	A0A7I2V2X8	Quercetin	5,280,343	− 7.9	ARG-381,ARG-355,PHE-356,R-359,ILE-406,HIS-362,GLU-360
DPP4	A0A7I2V2X8	Glycocholic acid	10,140	− 8.3	GLU-204,TYR-665,ASN-709,ARG-124,TYR-546,ASN-561,SER-629
CYP3A4	A0A494C192	Equol	91,469	− 7.6	SER-35,THR-34
CYP3A4	A0A494C192	Genistein	5,280,961	− 7.5	ARG-59,TYR-154,LEU-20,SER-162
CYP3A4	A0A494C192	Quercetin	5,280,343	− 8.3	TYR-154,GLN-331,SER-158,BAL-336
CYP3A4	A0A494C192	Glycocholic acid	10,140	− 8.8	GLU-155,GLN-331,LEU-58,LEU-329
EP300	Q09472	Equol	91,469	− 7.7	ARG-1462,SER-1400,LEU-1398
EP300	Q09472	Genistein	5,280,961	− 8.3	ASP-1399,GLN-1455,ARG-1410
EP300	Q09472	Quercetin	5,280,343	− 8.4	GLN-1455
EP300	Q09472	Glycocholic acid	10,140	− 7.5	LYS-1167,GLU-1169,ARG-1187
MGAM	O43451	Equol	91,469	− 7.8	ARG-48
MGAM	O43451	Genistein	5,280,961	− 8.5	ARG-48,GLN-32,THR-31,GLY-239
MGAM	O43451	Quercetin	5,280,343	− 8.4	ARG-48,GLN-32,THR-31,ARG-181,ASP-183
MGAM	O43451	Glycocholic acid	10,140	− 9.7	TYR-63,ARG-6,ASP-29,TYR-27,GLN-32,ARG-48
NR1H4	Q96RI1	Equol	91,469	− 9	SER-336,HIS-298
NR1H4	Q96RI1	Genistein	5,280,961	− 9	SER-336,HIS-298,TYR-365
NR1H4	Q96RI1	Quercetin	5,280,343	− 9	HIS-298
NR1H4	Q96RI1	Glycocholic acid	10,140	− 8.2	ASN-448,TYR-401

### Molecular dynamics simulation results

**Figure 10:**
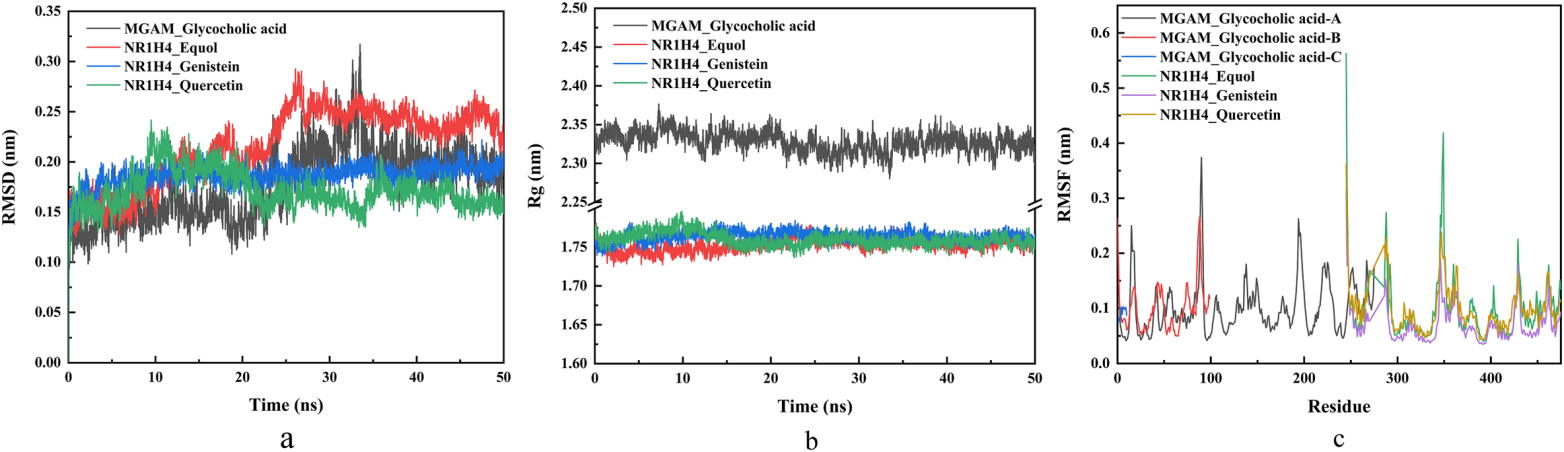
Molecular dynamics simulation results. (**a**) RMSD plot during molecular dynamics simulations. The black curve represents MGAM-glycocholic acid. The red curve represents NR1H4-equol. The blue curve represents NR1H4-genistein. The green curve represents NR1H4-quercetin. (**b**) Rog plot during molecular dynamics simulations. The black curve represents MGAM-glycocholic acid. The red curve represents NR1H4-equol. The blue curve represents NR1H4-genistein. The green curve represents NR1H4-quercetin. (**c**) RMSF plot during molecular dynamics simulations. The black curve represents MGAM-glycocholic acid-A. The red curve represents MGAM-glycocholic acid-B. The blue curve represents MGAM-glycocholic acid-C. The green curve represents NR1H4-equol. The purple curve represents NR1H4-genistein. The orange curve represents NR1H4-quercetin.

The root mean square deviation (RMSD) curve represents the fluctuation of protein conformation. It can be seen from the figure that in the beginning, RMSD increases because of the interactions between the complex and the solvent. Therefore, RMSD has certain fluctuations in the early stage. However, MGAM-glycocholic acid, NR1H4-equol, NR1H4-genistein and NR1H4-quercetin all increased briefly and tended to be stable, which indicated that the conformation of proteins would not change significantly after the combination of small molecular ligands with proteins, and the combination was relatively stable (Fig. 10a).

The gyration radius is often used to describe the change in the overall structure of a protein and to show the compactness of the overall structure. It can be seen from the figure that MGAM-glycocholic acid, NR1H4-equol, NR1H4-genistein and NR1H4-quercetin all have very stable gyration radii. This result is consistent with the RMSD curve reaction, which proves the stability of the protein conformation (Fig. 10b).

Root mean square fluctuation (RMSF) represents the fluctuation of the protein amino acid residues. This reflects the protein's flexibility in the molecular dynamics simulation process. Usually, after the drug is combined with the protein, the flexibility of the protein is reduced to stabilize the protein and play the role of enzyme. As seen from the figure, the simulation results of NR1H4 protein and equol drug, NR1H4 protein and genistein drug, and NR1H4 protein and quercetin drug show that the protein has good flexibility (Fig. 10c).

### Evaluation of drug similarity and toxicity characteristics

Using the SwissADME and ADMETlab platforms and according to Lipinski's Five Laws, the similarity and toxicity of equol, genistein, quercetin and glycocholic acid were evaluated, including molecular weight (≤ 500), H-bond receptor (≤ 10), H-bond donor (≤ 5), MlogP (≤ 4.15), bioavailability score (> 0.1), and topological polar surface area (< 140). The results showed that equol, genistein, quercetin and glycocholic acid could be accepted as new drugs based on pharmacokinetic parameters (Table 2).

**Table 2:** Physicochemical properties.

No	Compound	Lipinski rules			Moriguchi octanol–water partition coefficient	Lipinski’s violations	Bioavailability score	Topological surfacearea
		Molecular weight	Hydrogen bonding acceptor	Hydrogen bonding donor				
1	Equol	242	3	2	2.2	0	0.55	49.69
2	Genistein	270	5	3	0.52	0	0.55	90.9
3	Quercetin	302	7	5	− 0.56	0	0.55	131.36
4	Glycocholic acid	465	7	5	1.369	0	0.56	127.09

Despite an acceptable therapeutic value, a drug is still not acceptable as a final product if it exhibits unintended toxicity. Therefore, drug candidates should exceed toxicity limits for further validation. Therefore, equol, genistein, and quercetin were evaluated by the ADMETlab platform for hERG blockers, acute oral toxicity in rats, eye corrosion, and respiratory toxicity (including LD50 [5.238 mg/kg]). The results showed that these substances could play an important role in the treatment of depression (Table 3).

**Table 3:** Toxicity profile.

Compound	Equol	Genistein	Quercetin	Glycocholic acid
HERG blocker	Nor-blocker	Nor-blocker	Nor-blocker	Nor-blocker
Rat oral acute toxicity	Negative	Negative	Negative	Negative
Eye corrosion	Negative	Negative	Negative	Negative
Respiratory toxicity	Negative	Negative	Negative	Negative
LD50	5.238	5.632	5.331	5.173

## Discussion

Studies have shown that metabolites of intestinal microbes can play an essential role in a variety of psychiatric diseases via the “GMB” axis^33,34^.

In the PPI network, DPP4, CYP3A4, EP300 MGAM and NR1H4 showed higher degrees and were identified as essential genes of intestinal microbiota influencing the occurrence of depression through the brain–gut axis. The primary mechanism of action of DPP4 is the selective cleavage of cytokines and glucagon-like peptide-1^35^. Previous studies have found that DPP4 inhibitors can improve cognitive function and mitochondrial function in the brain^36^. Clinical studies have also confirmed the hypothesis that low plasma DPP4 activity is a characteristic marker of major depression and that changes in DPP4 enzyme activity play a role in the pathophysiology of major depression^37^. CYP3A4 is a metabolic enzyme widely found in human tissues and organs, is involved in approximately 50% of drug metabolism and is an essential factor affecting drug metabolism and efficacy in vivo^38^. Recent studies have confirmed that the mechanism of action of most antidepressants is related to the regulation of CYP3A4^39–43^. MGAM was associated with an increased risk of Alzheimer’s disease (AD) and major depressive disorder (MDD)^44,45^. NR1H4 is closely related to cholestasis, which can cause depression^46,47^. Cytochrome P450 oxidoreductase (POR) is involved in the biosynthesis of endogenous substances, such as bile acids and other steroids, as well as in the oxidative metabolism of xenobiotics, and POR knockdown resulted in the downregulation of NR1H4 (FXR) and the deregulation of bile acid and cholesterol biosynthesis^48^.

GO and KEGG enrichment analyses of hub genes showed that genes were primarily enriched in carbohydrate digestion and absorption, bile secretion, drug metabolism—cytochrome P450, and other pathways. Cytochrome P450 is one of the essential enzymes in drug oxidation metabolism because it can oxidize and metabolize many exogenous substances, including drugs. Secondary bile acids correspond to the metabolism of these products by the gut microbiome. The two primary BAs are cholic acid and deoxycholic acid, which are often secreted into bile in combination with taurine or glycine^49^. Activation of farnesoid X receptor (FXR) may play a central role in the onset of depression under pathological conditions^50^. During prenatal brain development, synapses form between neurons, resulting in neural circuits that support complex cognitive functions. Selective serotonin reuptake inhibitors are commonly used throughout pregnancy to treat depression^51^. Dysregulation of the serotonergic system has been reported to have a significant role in several neurological disorders, including depression, autism and substance abuse disorders^52^. Cholestasis can impair social motivation behavior and induce depression-like behavior. Cholestasis can also affect anxiety and pain behaviors in mice^53^. Pharmacotherapy for neuropsychiatric disorders, such as anxiety and depression, has been characterized by significant interindividual variability in drug response and the development of side effects. Pharmacogenetic research on depression and anxiety has focused on genetic polymorphisms affecting metabolism via cytochrome P450 (CYP)^54^. In antidepressant drug treatment, most drugs are metabolized via the polymorphic cytochrome P450 enzyme CYP2D6^55^. Activating cAMP-PKA signaling could prevent the behavioral changes and hippocampal synaptic deficits elicited by posttraumatic stress disorder (PTSD)^56^. Restoring hippocampal cAMP and BDNF levels could be an antidepressant treatment^57^. Decreases in cAMP and ERK1/2 phosphorylation could reduce the immobility time of chronic restraint stress (CRS) mice in the FST^58^. Research has found that regulating the HIF-1 signaling pathway can improve LPS-induced depressive behavior^59^, and CPSP with comorbid anxiety and depression can be improved by increasing cerebral blood flow and inhibiting HIF-1α/NLRP3 inflammatory signaling^60^.

According to the network analysis of “intestinal microbiota–metabolites–substrate–target genes”, equol, genistein, quercetin and glycocholic acid were found to be the key metabolites affecting depression. Studies have shown that intestinal microbiota such as *Enterococcus casseliflavus* and *Bacteroides* sp. 45 can metabolize the flavonoid quercetin-3-glucoside to produce quercetin^61,62^. Quercetin acts as an antidepressant by regulating neurotransmitter levels, promoting hippocampal neuron regeneration, reducing inflammation and antioxidant stress, and increasing serotonin levels^63,64^. Equol, a key metabolite of isoflavone with estrogenic and antioxidant activities^65^, can decrease body weight, abdominal WAT, and depression-related behaviors^66^. Experimental studies have found that S-equol can help reduce depression and anxiety in individuals^67^. Genistein, which is produced by the strains *“Hugonella massiliensis”* DSM 101782^T^ and “*Senegalimassilia faecalis”* KGMB04484^T^^68^, treats depression by suppressing the expression of miR-221/222 by targeting connexin^69^.

Molecular docking showed that the key metabolites had good binding activity with the hub genes, and the binding sites were hydrogen bonded to form a stable conformation, indicating that the combination of intestinal microbiota metabolites and depression targets may help in treating depression. Additionally, the molecular dynamics simulation results showed that MGAM-glycocholic acid, NR1H4-equol, NR1H4-genistein and NR1H4-quercetin bind stably.

Drug similarity and toxicity evaluations of equol, genistein, quercetin, and other metabolites revealed that they have antidepressant effects. Genistein can be found in *Pueraria lobata*, *Tempeh tempeh*, and *Cistanche deserticola*.

## Conclusions

In this study, we developed a comprehensive strategy to analyze the metabolites of the intestinal microbiota and the target genes of the intestinal microbiota affecting depression through systems biology. We explored the potential targets and inhibitors of the intestinal microbiota in treating depression. We found that intestinal microbiota metabolites such as quercetin, equol, and glycocholic acid can affect the course of depression by acting on targets such as MGAM and NR1H4. The mechanisms of action are related to carbohydrate digestion and absorption, bile secretion, drug metabolism-cytochrome P450, and other pathways. The mechanism of action has multitarget and multipathway results. Subsequently, we further verified these results by molecular docking and molecular dynamics simulations. Finally, we also evaluated the critical metabolites' drug similarity and toxicity characteristics to further confirm their potential drug possibilities. However, accumulating microbiome information has certain limitations, and we plan to conduct further preclinical or clinical trials to provide more references for its clinical application and development.

## Supplementary Information

Supplementary Table S1.

Supplementary Table S2.

Supplementary Table S3.

Supplementary Table S4.

Supplementary Table S5.

Supplementary Table S6.

Supplementary Table S7.

Supplementary Table S8.

## Data Availability

The depression data supporting this study's findings are available in the GEO database (http://www.ncbi.nlm.nih.gov/geo), reference numbers [GSE19738, GSE76826, and GSE182193], and the intestinal microbiota data can be found in the GutMgene database (http://bioannotation.cn/gutmgene/home.dhtml).

## Abbreviations

SEA: Similarity ensemble approach
STP: SwissTargetPrediction
GEO: Gene Expression Omnibus
DEGs: Differentially expressed genes
GO: Gene ontology
KEGG: Kyoto encyclopedia of genes and genomes
PPI: Protein‒protein interaction
MD: Molecular dynamics
ACPYPE: AnteChamber PYthon Parser interface
NVT: Canonical ensemble, constant pressure
NPT: Constant temperature
MF: Molecular functions
BP: Biological processes
CC: Cellular components
DPP4: Dipeptidyl peptidase 4
CYP3A4: Cytochrome P450 3A4
EP300: Histone acetyltransferase p300
MGAM: Maltase-glucoamylase
NR1H4: Bile acid receptor
RMSD: Root mean square deviation
RMSF: Root mean square fluctuation
FXR: Farnesoid X receptor

## References

[1] Dean J, Keshavan M (2017). The neurobiology of depression: An integrated view. Asian J. Psychiatry.

[2] Ng M, Fleming T, Robinson M (2014). Global, regional, and national prevalence of overweight and obesity in children and adults during 1980–2013: A systematic analysis for the Global Burden of Disease Study 2013. Lancet.

[3] Moreno-Agostino D, Wu YT, Daskalopoulou C (2021). Global trends in the prevalence and incidence of depression: A systematic review and meta-analysis. J. Affect. Disord..

[4] Plana-Ripoll O, Pedersen CB, Agerbo E (2019). A comprehensive analysis of mortality-related health metrics associated with mental disorders: A nationwide, register-based cohort study. Lancet.

[5] Tran BX, Ha GH, Nguyen DN (2020). Global mapping of interventions to improve quality of life of patients with depression during 1990–2018. Qual. Life Res..

[6] Malhi GS, Mann JJ (2018). Depression. Lancet.

[7] Trivedi MH, Rush AJ, Wisniewski SR (2006). Evaluation of outcomes with citalopram for depression using measurement-based care in STAR*D: Implications for clinical practice. Am. J. Psychiatry.

[8] Li Z, Zhu H, Zhang L, Qin C (2018). The intestinal microbiome and Alzheimer's disease: A review. Anim. Models Exp. Med..

[9] Choi HH, Cho YS (2016). Fecal microbiota transplantation: Current applications, effectiveness, and future perspectives. Clin. Endosc..

[10] Wang Y, Kasper LH (2014). The role of microbiome in central nervous system disorders. Brain Behav. Immun..

[11] Farzi A, Hassan AM, Zenz G, Holzer P (2019). Diabesity and mood disorders: Multiple links through the microbiota-gut-brain axis. Mol. Asp. Med..

[12] Aizawa E, Tsuji H, Asahara T (2016). Possible association of *Bifidobacterium* and *Lactobacillus* in the gut microbiota of patients with major depressive disorder. J. Affect. Disord..

[13] Luna RA, Foster JA (2015). Gut brain axis: Diet microbiota interactions and implications for modulation of anxiety and depression. Curr. Opin. Biotechnol..

[14] Jiang H, Ling Z, Zhang Y (2015). Altered fecal microbiota composition in patients with major depressive disorder. Brain Behav. Immun..

[15] Mayer EA, Knight R, Mazmanian SK, Cryan JF, Tillisch K (2014). Gut microbes and the brain: Paradigm shift in neuroscience. J. Neurosci..

[16] Cruz-Pereira JS, Rea K, Nolan YM (2020). Depression's unholy trinity: Dysregulated stress, immunity, and the microbiome. Annu. Rev. Psychol..

[17] Kelly JR, Borre Y, O’Brien C (2016). Transferring the blues: Depression-associated gut microbiota induces neurobehavioural changes in the rat. J. Psychiatr. Res..

[18] Cheng L, Qi C, Yang H (2022). gutMGene: A comprehensive database for target genes of gut microbes and microbial metabolites. Nucleic Acids Res..

[19] Keiser MJ, Roth BL, Armbruster BN (2007). Relating protein pharmacology by ligand chemistry. Nat. Biotechnol..

[20] Gfeller D, Michielin O, Zoete V (2013). Shaping the interaction landscape of bioactive molecules. Bioinformatics.

[21] Davis S, Meltzer PS (2007). GEOquery: A bridge between the Gene Expression Omnibus (GEO) and BioConductor. Bioinformatics.

[22] Wickham, H. *ggplot2: Elegant Graphics for Data Analysis* 1 (2016).

[23] Kanehisa M, Furumichi M, Tanabe M, Sato Y, Morishima K (2017). KEGG: New perspectives on genomes, pathways, diseases and drugs. Nucleic Acids Res..

[24] Shannon P, Markiel A, Ozier O (2003). Cytoscape: A software environment for integrated models of biomolecular interaction networks. Genome Res..

[25] Otasek D, Morris JH, Boucas J, Pico AR, Demchak B (2019). Cytoscape automation: Empowering workflow-based network analysis. Genome Biol..

[26] Trott O, Olson AJ (2010). AutoDock Vina: Improving the speed and accuracy of docking with a new scoring function, efficient optimization, and multithreading. J. Comput. Chem..

[27] Jumper J, Evans R, Pritzel A (2021). Highly accurate protein structure prediction with AlphaFold. Nature.

[28] Varadi M, Anyango S, Deshpande M (2022). AlphaFold protein structure database: Massively expanding the structural coverage of protein-sequence space with high-accuracy models. Nucleic Acids Res..

[29] Hess B, Kutzner C, van der Spoel D, Lindahl E (2008). GROMACS 4: Algorithms for highly efficient, load-balanced, and scalable molecular simulation. J. Chem. Theory Comput..

[30] Lindorff-Larsen K, Piana S, Palmo K (2010). Improved side-chain torsion potentials for the Amber ff99SB protein force field. Proteins.

[31] Daina A, Michielin O, Zoete V (2017). SwissADME: A free web tool to evaluate pharmacokinetics, drug-likeness and medicinal chemistry friendliness of small molecules. Sci. Rep..

[32] Dong J, Wang NN, Yao ZJ (2018). ADMETlab: A platform for systematic ADMET evaluation based on a comprehensively collected ADMET database. J. Cheminform..

[33] Hills RJ, Pontefract BA, Mishcon HR (2019). Gut microbiome: Profound implications for diet and disease. Nutrients.

[34] Asadi A, Shadab MN, Mohamadi MH (2022). Obesity and gut-microbiota-brain axis: A narrative review. J. Clin. Lab. Anal..

[35] Taylor SI, Yazdi ZS, Beitelshees AL (2021). Pharmacological treatment of hyperglycemia in type 2 diabetes. J. Clin. Invest..

[36] Pipatpiboon N, Pintana H, Pratchayasakul W, Chattipakorn N, Chattipakorn SC (2013). DPP4-inhibitor improves neuronal insulin receptor function, brain mitochondrial function and cognitive function in rats with insulin resistance induced by high-fat diet consumption. Eur. J. Neurosci..

[37] Maes M, De Meester I, Scharpe S (1996). Alterations in plasma dipeptidyl peptidase IV enzyme activity in depression and schizophrenia: Effects of antidepressants and antipsychotic drugs. Acta Psychiatr. Scand..

[38] Liu YT, Hao HP, Liu CX, Wang GJ, Xie HG (2007). Drugs as CYP3A probes, inducers, and inhibitors. Drug Metab. Rev..

[39] Kot M, Haduch A, Papp M, Daniel WA (2017). The effect of chronic treatment with lurasidone on rat liver cytochrome P450 expression and activity in the chronic mild stress model of depression. Drug Metab. Dispos..

[40] Andrade C (2017). Ketamine for depression, 5: Potential pharmacokinetic and pharmacodynamic drug interactions. J. Clin. Psychiatry.

[41] Chen L, Boinpally R, Gad N (2015). Evaluation of cytochrome P450 (CYP) 3A4-based interactions of levomilnacipran with ketoconazole, carbamazepine or alprazolam in healthy subjects. Clin. Drug Investig..

[42] Ghosh C, Hossain M, Spriggs A (2015). Sertraline-induced potentiation of the CYP3A4-dependent neurotoxicity of carbamazepine: An in vitro study. Epilepsia.

[43] Menus A, Kiss A, Toth K (2020). Association of clozapine-related metabolic disturbances with CYP3A4 expression in patients with schizophrenia. Sci. Rep..

[44] Meng L, Wang Z, Ji HF, Shen L (2022). Causal association evaluation of diabetes with Alzheimer's disease and genetic analysis of antidiabetic drugs against Alzheimer's disease. Cell Biosci..

[45] Huang SS, Chen YT, Su MH (2023). Investigating genetic variants for treatment response to selective serotonin reuptake inhibitors in syndromal factors and side effects among patients with depression in Taiwanese Han population. Pharmacogenom. J..

[46] Khakpai F, Rezaei N, Issazadeh Y, Zarrindast MR (2023). Modulation of social and depression behaviors in cholestatic and drug-dependent mice: Possible role of opioid receptors. J. Diabetes Metab. Disord..

[47] Deng F, Qin G, Chen Y (2023). Multi-omics reveals 2-bromo-4,6-dinitroaniline (BDNA)-induced hepatotoxicity and the role of the gut-liver axis in rats. J. Hazard. Mater..

[48] Heintze T, Wilhelm D, Schmidlin T (2021). Effects of diminished NADPH: Cytochrome P450 reductase in human hepatocytes on lipid and bile acid homeostasis. Front. Pharmacol..

[49] Chiang JY (2013). Bile acid metabolism and signaling. Compr. Physiol..

[50] Trzeciak P, Herbet M (2021). Role of the intestinal microbiome, intestinal barrier and psychobiotics in depression. Nutrients.

[51] Tate K, Kirk B, Tseng A, Ulffers A, Litwa K (2021). Effects of the selective serotonin reuptake inhibitor fluoxetine on developing neural circuits in a model of the human fetal cortex. Int. J. Mol. Sci..

[52] Chaji D, Venkatesh VS, Shirao T, Day DJ, Ellenbroek BA (2021). Genetic knockout of the serotonin reuptake transporter results in the reduction of dendritic spines in in vitro rat cortical neuronal culture. J. Mol. Neurosci..

[53] Khakpai F, Issazadeh Y, Rezaei N, Zarrindast MR (2022). Enhanced anxiolytic and analgesic effectiveness or a better safety profile of morphine and tramadol combination in cholestatic and addicted mice. NeuroReport.

[54] Radosavljevic M, Svob SD, Jancic J, Samardzic J (2023). The role of pharmacogenetics in personalizing the antidepressant and anxiolytic therapy. Genes (Basel).

[55] Kirchheiner J, Seeringer A (2007). Clinical implications of pharmacogenetics of cytochrome P450 drug metabolizing enzymes. Biochim. Biophys. Acta.

[56] Ji M, Zhang Z, Gao F (2023). Curculigoside rescues hippocampal synaptic deficits elicited by PTSD through activating cAMP-PKA signaling. Phytother. Res..

[57] Zaki ES, Sayed RH, Saad MA, El-Yamany MF (2023). Roflumilast ameliorates ovariectomy-induced depressive-like behavior in rats via activation of AMPK/mTOR/ULK1-dependent autophagy pathway. Life Sci..

[58] Oh DR, Choi C, Kim MJ (2023). Antidepressant effects of p-coumaric acid isolated from *Vaccinium bracteatum* leaves extract on chronic restraint stress mouse model and antagonism of serotonin 6 receptor in vitro. Phytomedicine.

[59] Li A, Liu Z, Ali T (2023). Roxadustat (FG-4592) abated lipopolysaccharides-induced depressive-like symptoms via PI3K signaling. Front. Mol. Neurosci..

[60] Shi ZM, Jing JJ, Xue ZJ (2023). Stellate ganglion block ameliorated central post-stroke pain with comorbid anxiety and depression through inhibiting HIF-1alpha/NLRP3 signaling following thalamic hemorrhagic stroke. J. Neuroinflamm..

[61] Chen S, Tang Y, Gao Y (2022). Antidepressant potential of quercetin and its glycoside derivatives: A comprehensive review and update. Front. Pharmacol..

[62] Shen Z, Xu Y, Jiang X (2019). Avicularin relieves depressive-like behaviors induced by chronic unpredictable mild stress in mice. Med. Sci. Monit..

[63] Bax EN, Cochran KE, Mao J, Wiedmeyer CE, Rosenfeld CS (2019). Opposing effects of S-equol supplementation on metabolic and behavioral parameters in mice fed a high-fat diet. Nutr. Res..

[64] Schneider H, Simmering R, Hartmann L, Pforte H, Blaut M (2000). Degradation of quercetin-3-glucoside in gnotobiotic rats associated with human intestinal bacteria. J. Appl. Microbiol..

[65] Yang J, Qian D, Jiang S (2012). Identification of rutin deglycosylated metabolites produced by human intestinal bacteria using UPLC-Q-TOF/MS. J. Chromatogr. B Analyt. Technol. Biomed. Life Sci..

[66] Mayo B, Vazquez L, Florez AB (2019). Equol: A bacterial metabolite from the daidzein isoflavone and its presumed beneficial health effects. Nutrients.

[67] Blake C, Fabick KM, Setchell KD, Lund TD, Lephart ED (2011). Neuromodulation by soy diets or equol: Anti-depressive & anti-obesity-like influences, age- & hormone-dependent effects. BMC Neurosci..

[68] Soukup ST, Stoll DA, Danylec N (2021). Metabolism of daidzein and genistein by gut bacteria of the class *Coriobacteriia*. Foods.

[69] Shen F, Huang WL, Xing BP (2018). Genistein improves the major depression through suppressing the expression of miR-221/222 by targeting connexin 43. Psychiatry Investig..

